# Mechanism of hepatotoxicity induced by ethanol extract of *Emilia sonchifolia* (L.) DC revealed by proteomics and metabolomics

**DOI:** 10.3389/fphar.2025.1669607

**Published:** 2025-11-10

**Authors:** Gongzhen Chen, Qiantonghan Luo, Zicong Song, Ping Zheng, Xin Liu, Ting Tang

**Affiliations:** 1 Guizhou University of Traditional Chinese Medicine, Guiyang, China; 2 The First Affiliated Hospital of Guizhou University of Traditional Chinese Medicine, Guiyang, China

**Keywords:** drug-induced liver injury, ethanol extract of Emilia sonchifolia (L.) DC, hepatotoxicity, proteomics, metabolomics

## Abstract

**Objective:**

Hepatotoxicity has been reported for *Emilia sonchifolia* (L.) DC (*E. sonchifolia*). The plant material is typically prepared using two extraction methods for practical application: water extraction and ethanol extraction. However, our previous research only investigated its water extract. Therefore, this study aims to systematically evaluate the hepatotoxicity and underlying mechanisms of the ethanol extract of *E. sonchifolia,* thereby providing a more comprehensive scientific basis for its rational application and safety assessment.

**Methods:**

An acute toxicity preliminary screening study was conducted by orally administering *E. sonchifolia* ethanol extract to mice at doses ranging from 0 to 33.6 g/kg/day. Based on the results of the acute toxicity test preliminary screening study, mice were divided into a control group and an *E. sonchifolia* ethanol extract group (8.6 g/kg/day) for a 14-day delayed hepatotoxicity experiment based on clinical treatment duration. At the end of the intervention, hepatic pathological changes were examined using hematoxylin-eosin staining. Enzyme-linked immunosorbent assay (ELISA) was employed to quantify the levels of alanine aminotransferase, aspartate aminotransferase, total bilirubin, direct bilirubin, total bile acids, alkaline phosphatase, and γ-glutamyl transferase in serum, as well as malondialdehyde, superoxide dismutase, and catalase in liver tissue. Proteomics and metabolomics analyses were performed to investigate the mechanisms of hepatotoxicity induced by the ethanol extract. Additionally, the mRNA expression levels of *Cyp3a41a*, *Cyp2c29*, *Ugt2b1*, and *Hsd3b3* in mice liver tissue were determined using quantitative reverse transcription polymerase chain reaction (qRT-PCR).

**Results:**

The acute toxicity preliminary screening study results showed that a dose of 12.0 g/kg or higher of the *E. sonchifolia* ethanol extract caused acute liver failure and death in mice. A dose of 8.6 g/kg or lower of the *E. sonchifolia* ethanol extract produced dose-dependent acute hepatotoxicity. Meanwhile, a dose of 8.6 g/kg of the *E. sonchifolia* ethanol extract induced delayed toxicities in mice. Proteomics and metabolomics results revealed that the hepatotoxicity induced by the ethanol extract of *E. sonchifolia* was associated with cholestasis and oxidative stress caused by disruptions in drug metabolism, steroid hormone biosynthesis, and primary bile acid biosynthesis. Validation experiments showed that the levels of *Cyp2c29* were decreased, while the mRNA levels of *Cyp3a41a, Ugt2b1*, and *Hsd3b3* were increased in the liver tissues of mice treated with the ethanol extract of *E. sonchifolia*. Additionally, serum levels of total bilirubin, direct bilirubin, total bile acids, alkaline phosphatase, and γ-glutamyl transferase were significantly elevated. Furthermore, in the livers of mice treated with the ethanol extract, malondialdehyde levels were increased, whereas superoxide dismutase and catalase levels were decreased.

**Conclusion:**

In summary, the ethanol extract of *E. sonchifolia* can induce hepatotoxicity in mice, and its mechanism is associated with cholestasis and oxidative stress mediated by disruptions in drug metabolism, steroid hormone biosynthesis, and primary bile acid biosynthesis.

## 1 Introduction

In recent years, traditional Chinese medicine has gained widespread use globally due to its notable therapeutic effects (Ma et al., 2023). However, its application has been associated with various adverse effects, including hepatotoxicity, nephrotoxicity, cardiotoxicity, neurotoxicity, and carcinogenicity (Ma et al., 2023; Zhai et al., 2021). Among these, the liver, as the primary organ responsible for drug metabolism, is particularly vulnerable to injury due to the potential generation of hepatotoxic metabolites during metabolism (Bjornsson and Jonasson, 2013). Consequently, herb-induced liver injury has emerged as a prevalent and concerning adverse reaction. A systematic review and meta-analysis revealed that the mortality rate associated with herb-related liver injury can be as high as 10.4% (Singh et al., 2015). These findings underscore that herb-induced liver injury represents a significant public health concern requiring urgent attention.

*Emilia sonchifolia* (L.) DC (*E. sonchifolia*), a widely used medicinal and edible herb, possesses therapeutic properties such as clearing heat and detoxifying, activating blood circulation to remove stasis, and reducing swelling and alleviating pain (Yu et al., 2021). The plant name has been checked with “World Flora Online” (www.worldfloraonline.org) mentioning the data of accessing that website. It is commonly employed in the treatment of various diseases, including upper respiratory tract infections, oral ulcers, pneumonia, mastitis, enteritis, bacillary dysentery, and urinary tract infections (George and Kuttan, 2019; Luo et al., 2019; Urumbil and Anilkumar, 2021). Despite its significant efficacy in traditional medicine, the potential adverse effects of *E. sonchifolia* cannot be overlooked. Clinical reports have documented cases of hepatic sinusoidal obstruction syndrome caused by *E. sonchifolia* (Deng et al., 2021). This has greatly limited the application and broader promotion of the herb and its related preparations.

*Emilia sonchifolia* is typically prepared using either water or ethanol extraction (An et al., 2025; Liu et al., 2025). Ethanol extraction can preserve constituents more completely than water extraction (Miao et al., 2019; Yang et al., 2022), which may result in higher levels of toxic components and stronger hepatotoxicity. In our previous study, the hepatotoxic mechanisms were explored only for the aqueous extract. To provide a more comprehensive scientific basis for the rational use and safety assessment of *E. sonchifolia*, the present study further investigates the hepatotoxicity of its ethanol extract.

Omics technologies have been widely applied to explore the molecular complexity of biological systems and have shown great potential in elucidating the mechanisms of herb-induced hepatotoxicity (Chong et al., 2023; Gentien et al., 2023; Sathyanarayanan et al., 2023; Subramanian et al., 2020; Wang et al., 2023). By integrating multi-omics data, toxicological mechanisms of traditional herbal medicines can be analyzed from multiple dimensions (Canzler et al., 2020; Karkossa et al., 2020). Approaches such as proteomics and metabolomics provide valuable insights into comprehensively understanding the hepatotoxicity mechanisms of traditional herbal formulations.

This study confirmed the hepatotoxicity of *E. sonchifolia* ethanol extract through acute toxicity preliminary screening study and investigated its underlying mechanisms using proteomic and metabolomic analyses. The expression levels of target genes involved in *E. sonchifolia*-induced hepatotoxicity were validated via quantitative reverse transcription polymerase chain reaction (qRT-PCR), while cholestasis-related biomarkers and oxidative stress enzyme activities were quantified using enzyme-linked immunosorbent assay (ELISA). These findings offer a theoretical foundation for further in-depth studies and the development of therapeutic strategies to mitigate *E. sonchifolia*-induced hepatotoxicity.

## 2 Materials and methods

### 2.1 Plant material

Due to the higher bioactivity and lower risk of microbial contamination associated with ethanol extracts, this study focused on the ethanol extract of *E. sonchifolia*. The whole dried plant of *E. sonchifolia* was purchased from Shanxi Xibolan Biotechnology Co., Ltd. (Xian, Shanxi, China). The plant material was air-dried in a cool, shaded area and ground into a fine powder. A total of 500 g of *E. sonchifolia* powder was extracted twice with 30% ethanol. The combined extracts were concentrated using a rotary evaporator and dried in a vacuum drying oven. Finally, 109.5 g of ethanol extract dry powder was obtained.

### 2.2 Animals

The research was conducted in accordance with the internationally accepted principles for laboratory animal use and care as found in the US guidelines (NIH publication #85-23, revised in 1985). Specific pathogen free ICR mice (18–22 g, half male and half female) were purchased from Henan Skobes Biotechnology Co., Ltd. (Anyang, Henan, China) [License number: SCXK (Yu) 2020-0005]. The animals were housed in the specific pathogen free-grade animal facility at the Animal Research Center of Guizhou University of Traditional Chinese Medicine. Mice were separated by gender and maintained under controlled conditions with a temperature of 23 °C ± 2 °C, relative humidity of 40%–60%, and a 12-h light/12-h dark cycle. Food and water were provided *ad libitum*. After a 7-day acclimation period, the experiments were initiated. All experimental procedures and protocols were approved by the Animal Ethics Committee of Guizhou University of Traditional Chinese Medicine (Ethics Approval Number: 20230041).

### 2.3 Acute toxicity preliminary screening study

Fifty-four ICR mice were randomly assigned to 9 groups, each comprising 6 mice with an equal male-to-female ratio. Prior to the experiment, the mice were fasted and deprived of water for 12 h. Dosage groups were established at 0 g/kg, 4.4 g/kg, 6.2 g/kg, 8.6 g/kg, 12.0 g/kg, 17.0 g/kg, 24.0 g/kg, 27.0 g/kg, and 33.6 g/kg, along with a control group, based on the acute toxicity data of *E. sonchifolia* ethanol extract reported by Zhong et al. (2006)., as well as preliminary experimental results. Each dosage group received a single oral gavage of the corresponding dose of the ethanol extract, while the control group (0 g/kg) received an equivalent volume of phosphate buffer saline. Behavioral changes and survival were monitored for 24 h post-administration.

### 2.4 Delayed hepatotoxicity experiment

Twelve ICR mice were randomly assigned to two groups: the *E. sonchifolia* ethanol extract group and the control group, with 6 mice per group and an equal male-to-female ratio. The treatment group received *E. sonchifolia* ethanol extract at a dose of 8.6 g/kg/day, while the control group was administered phosphate buffer saline. The dosing regimen lasted for 14 consecutive days, which is consistent with the typical clinical administration duration. After the treatment period, the mice were euthanized with pentobarbital sodium (100 mg/kg, intraperitoneal injection), and blood and liver tissue samples were collected for subsequent analysis.

### 2.5 Hematoxylin and eosin staining

Liver tissue samples from the mice were subjected to gradient dehydration, clearing, paraffin embedding, sectioning, and dewaxing. The sections were then stained with Hematoxylin and Eosin. After staining, the sections were dehydrated, cleared, and mounted with a coverslip. Pathological changes in the liver tissue were observed under a light microscope.

### 2.6 Enzyme-linked immunosorbent assay (ELISA)

Blood samples were collected from mice and centrifuged to obtain serum. Alanine aminotransferase, aspartate aminotransferase, total bilirubin, direct bilirubin, total bile acid, alkaline phosphatase, and γ-glutamyl transferase levels were measured using ELISA kits provided by Changchun Huili Biotech Co., Ltd. (Changchun, Jilin, China). Liver tissues were excised, minced, and homogenized in phosphate-buffered saline (1:9 weight-to-volume ratio) on an ice bath using a tissue grinder. The homogenates were centrifuged, and the supernatants were collected. Malondialdehyde, superoxide dismutase and catalase levels were quantified using ELISA kits obtained from Nanjing Jiancheng Bioengineering lnstitute (Nanjing, Jiangsu, China).

### 2.7 Determination and analysis of proteomic and metabolomic

#### 2.7.1 Proteomic sequencing and analysis

Liver tissue samples were collected and immediately frozen in liquid nitrogen for proteomic analysis. TMT-based quantitative proteomics analyses were implemented as Zhang described (Zhang et al., 2023). Three biological replicates were prepared for ethanol extract control group for proteomics analysis. The specific operations will be carried out by LC-BIO Technologies Co., Ltd. (Hangzhou, Zhejiang, China). Detailed protein extraction, purification, peptide tagging and reverse-phase chromatography and mass spectrometry were referred to previous report (Zhang et al., 2023). Protein identification, quantification, classification and interaction prediction were analyzed (Liu et al., 2022). The raw files generated by AQ Exactive Plus were converted using Proteome Discoverer 2.1 (Thermo Fisher Scientific), and the files were sent to OmicStudio tools for analysis (Kolli et al., 2023). Differentially expressed proteins were defined as those with a fold change ≥1.2 or ≤1/1.2 -fold and a P-value<0.05. Protein-protein interaction was further analyzed by string (http://www.string-db.org) and the core genes were screened by cytoscape software. The differentially expressed proteins were functionally classified by Gene Ontology (GO) terms (http://www.omicsbean.com). The Kyoto Encyclopedia of Genes and Genomes (KEGG) pathway of altered proteins was categorized utilizing the same resource.

### 2.8 Metabolomic sequencing and analysis

Liver tissue samples were collected for metabolomic analysis. Untargeted metabolomics profiling was performed as Chen described (Chen et al., 2023). Six biological replicates were prepared for ethanol extract and control group for metabolomics analysis. The specific operations will be carried out by LC-BIO Technologies Co., Ltd. (Hangzhou, Zhejiang, China). Partial least squares discriminant analysis were employed to reveal differences between groups. KEGG enrichment analysis was performed on significantly different metabolites (fold change) ≥1.2 or ≤1/1.2 -fold and a P-value<0.05 and Variable importance in projection≥1). Human Metabolome Database (https://hmdb.ca/) was utilized to process and analyze metabolites, and metabolic changes and pathways were concluded. Pathway enrichment analysis was conducted using KEGG and Gene Set Enrichment Analysis to interpret the metabolic changes induced by *E. sonchifolia* ethanol extract.

### 2.9 Integrated proteomics and metabolomics analysis

The top 30 enriched proteins and metabolites were subjected to correlation network and heatmap analysis using the OmicStudio cloud platform (https://www.omicstudio.cn/). Venn diagrams were generated to identify shared pathways between proteomics and metabolomics enrichments using the Bioinformatics online analysis platform (www.bioinformatics.com.cn). Shared pathways were further visualized in bubble plots to display the enrichment status of these pathways. This integrated analysis provided a comprehensive understanding of the molecular mechanisms underlying *E. sonchifolia*-induced hepatotoxicity by linking protein alterations and metabolic changes.

### 2.10 Quantitative reverse transcription polymerase chain reaction (qRT-PCR)

Quantitative RT-PCR was used to detect the mRNA expression levels of *Cyp2c29*, *Cyp3a41a*, *Ugt2b1*, and *Hsd3b3* in mouse liver tissue. Total RNA was extracted from mouse liver tissue using tissue lysis and centrifugation methods. The reverse transcription reaction was performed in two steps: RT1 for the removal of genomic DNA, and RT2 for the preparation of the reverse transcription reaction mixture. The reaction conditions were as follows: initial denaturation at 95 °C for 10 min (1 cycle); denaturation at 95 °C for 15 s (40 cycles); annealing and extension at 60 °C for 60 s (40 cycles); melt curve collection at 95 °C for 15 s, 60 °C for 60 s, and 95 °C for 15 s (1 cycle). The qRT-PCR primer sequences are listed in Table 1, and all primers were synthesized by Beijing Tsingke Biotech Co., Ltd. (Beijing, China). Each containing three replicates for all genes and the relative fold changes were calculated using the 2^−ΔΔCT^ method.

**TABLE 1:** Information of primers.

Gene	Primer	Sequence (5′-3′)	PCR products
Mus *β-actin*	Forward	CCA​GCC​TTC​CTT​CTT​GGG​TAT	103 bp
	Reverse	GTT​GGC​ATA​GAG​GTC​TTT​ACG​G	
Mus *Cyp2c29*	Forward	GTG​AAG​AAC​ATC​AGC​CAA​TCC	207 bp
	Reverse	GCT​AAA​AAC​AAC​GCC​AAA​ACC	
Mus *Ugt2b1*	Forward	CTT​CTG​CTT​CCA​TCC​TCA​TT	122 bp
	Reverse	TGT​CCA​GTT​TCC​TAC​CCA​TT	
Mus *Cyp3a41a*	Forward	AAG​AGG​CAG​AGA​AAG​GCA​AG	190 bp
	Reverse	AGT​ACA​ACT​GAG​AAG​ACC​AA	
Mus *Hsd3b3*	Forward	GTG​TGC​CAG​CCT​TCA​TCT​TCT	149 bp
	Reverse	TGC​CTT​CTC​AGC​CAT​CTT​TTT	

### 2.11 Statistical analysis

Values were expressed as mean ± SEM. The statistical differences among the different groups were compared using two-sided Student’s t-test Values of *P* < 0.05 were considered statistically significant (*P < 0.05; **P < 0.01; ***P < 0.001). The statistical analysis was performed by GraphPad Prism version 10.0 software (GraphPad, San Diego, California, United States).

## 3 Results

### 3.1 The *Emilia sonchifolia* ethanol extract displayed acute toxicity in mice

**FIGURE 1:**
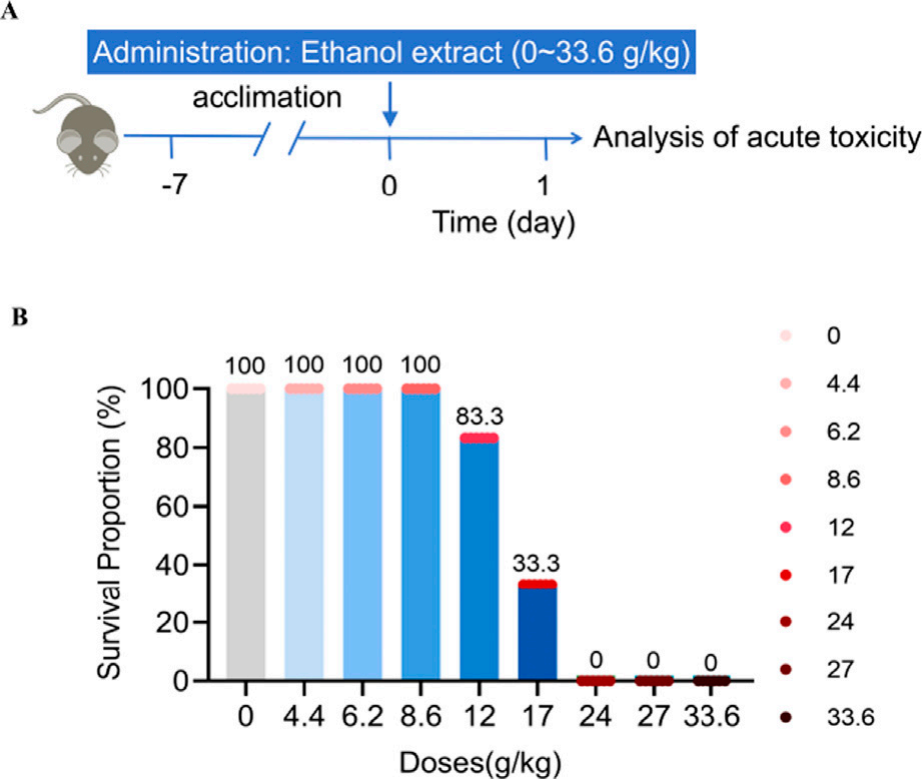
Acute toxicity evaluation of *Emilia sonchifolia* ethanol extract. **(A)** Experimental scheme determining the acute toxicity of *Emilia sonchifolia* ethanol extract in mice. **(B)** Effect of different doses of *Emilia sonchifolia* ethanol extract on mice survival (n = 6).

The acute toxicity preliminary screening study was conducted to assess the effects of different doses of *Emilia sonchifolia* ethanol extract on mice survival through continuous oral administration, ranging from 0 to 33.6 g/kg (Figure 1A). The results showed that the survival rate of the mice was 100% when they were given doses of 8.6 g/kg or below of *E. sonchifolia* ethanol extract. However, the survival rate dropped to 83.3% when the mice were administered a dose of 12.0 g/kg of the *E. sonchifolia* ethanol extract. When mice were given a *E. sonchifolia* ethanol extract at a dose of 17.0 g/kg, the survival rate was only 33.3%. However, when mice were administered doses equal to or greater than 24.0 g/kg of the same ethanol extract, the survival rate dropped to 0 (Figure 1B).

### 3.2 The *Emilia sonchifolia* ethanol extract induced delayed hepatotoxicity in mice

**FIGURE 2:**
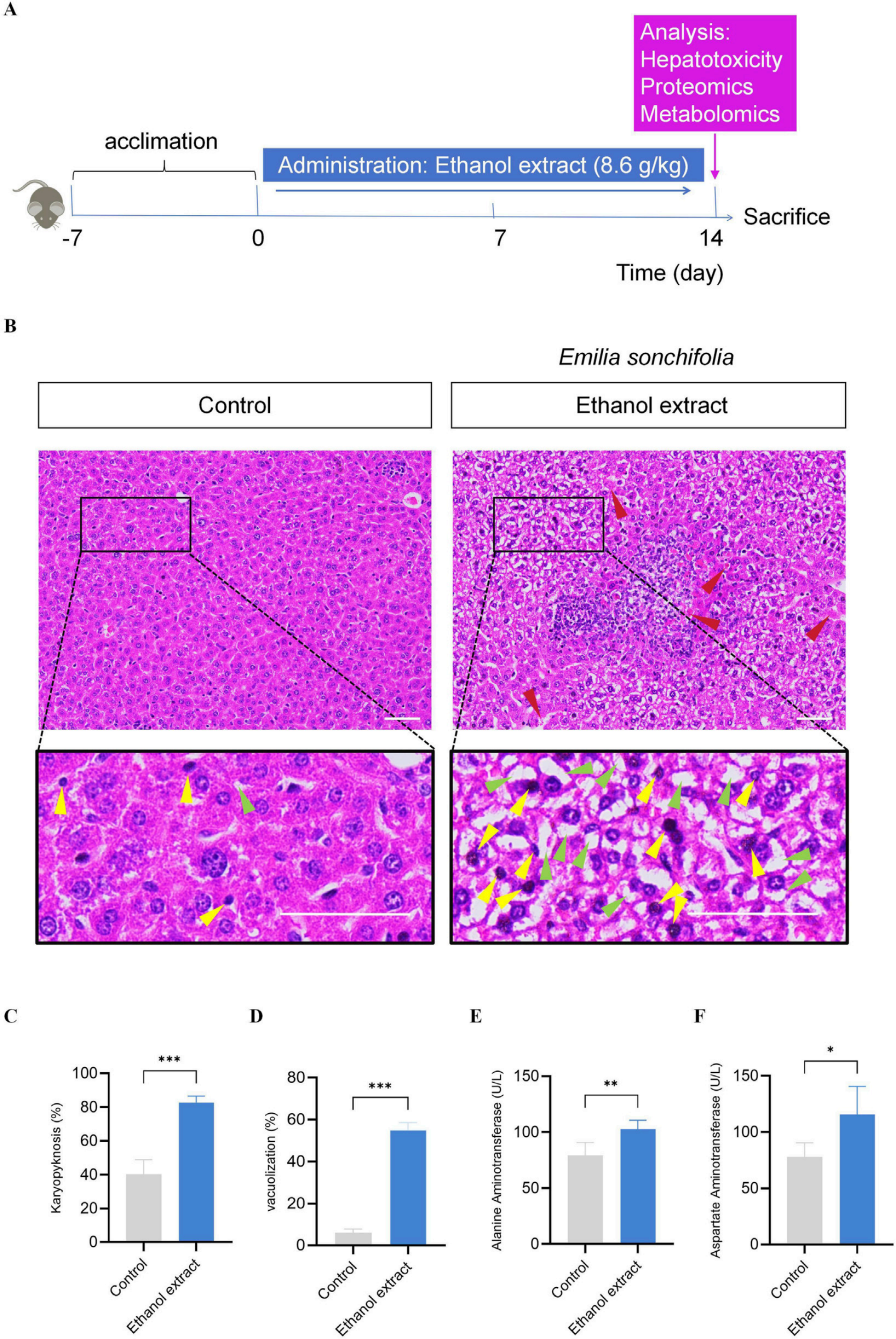
Investigation of the delayed hepatotoxicity of *Emilia sonchifolia* ethanol extract in mice. **(A)** The timeline for determining the delayed hepatotoxicity of Emilia sonchifolia ethanol extract in mice. **(B)** Representative micrograph of section of liver tissue from the mice treated with phosphate buffer saline or *Emilia sonchifolia* ethanol extract (8.6 g/kg). The bottom image is a magnification of the yellow box above. Yellow arrows represent hepatocytes with karyopyknosis. Green arrows represent vacuolated hepatocytes. Red arrows represent dilatation of the hepatic sinusoids around the central vein and increased spaces between hepatocytes. Scale bar, 50 μm. **(C,D)** Quantification of hepatocytes with karyopyknosis **(C)** and vacuolated hepatocytes **(D)** in liver tissue from the mice treated with phosphate buffer saline or *Emilia sonchifolia* ethanol extract. Four mice from each group were examined, and four hepatic micrographs (200 ×) from each animal were quantified. **(E,F)** Quantification of aspartate aminotransferase **(E)** and alanine aminotransferase **(F)** in serum from the mice treated with phosphate buffer saline or *Emilia sonchifolia* ethanol extract. Data are presented as mean ± SEM (n = 4). *p < 0.05, **p < 0.01, ***p < 0.001 by unpaired t-test.

Based on the results of the acute toxicity study, *E. sonchifolia* ethanol extract at a dose of 8.6 g/kg was selected to investigate its delayed hepatotoxicity and underlying mechanisms. (Figure 2A). Histopathological analysis of liver tissues revealed significantly increased nuclear pyknosis and vacuolation in the *E. sonchifolia* ethanol extract group compared to the control group (P < 0.001). Furthermore, the livers of mice in the *E. sonchifolia* ethanol extract group showed marked dilatation of the hepatic sinusoids around the central vein and increased spaces between hepatocytes, resembling the pathological findings of hepatic sinusoidal obstruction syndrome in mice (Zhu et al., 2022) (Figures 2B–D **)**. ELISA results indicated that alanine aminotransferase and aspartate aminotransferase levels were significantly elevated in the *E. sonchifolia* ethanol extract group compared to the control group (P < 0.01) (Figures 2E,F). These findings suggest that *E. sonchifolia* ethanol extract at a dose of 8.6 g/kg induces delayed hepatotoxicity in mice.

### 3.3 Comprehensive proteomics and metabolomics analysis reveals the mechanism of hepatotoxicity induced by *Emilia sonchifolia* ethanol extract

**FIGURE 3:**
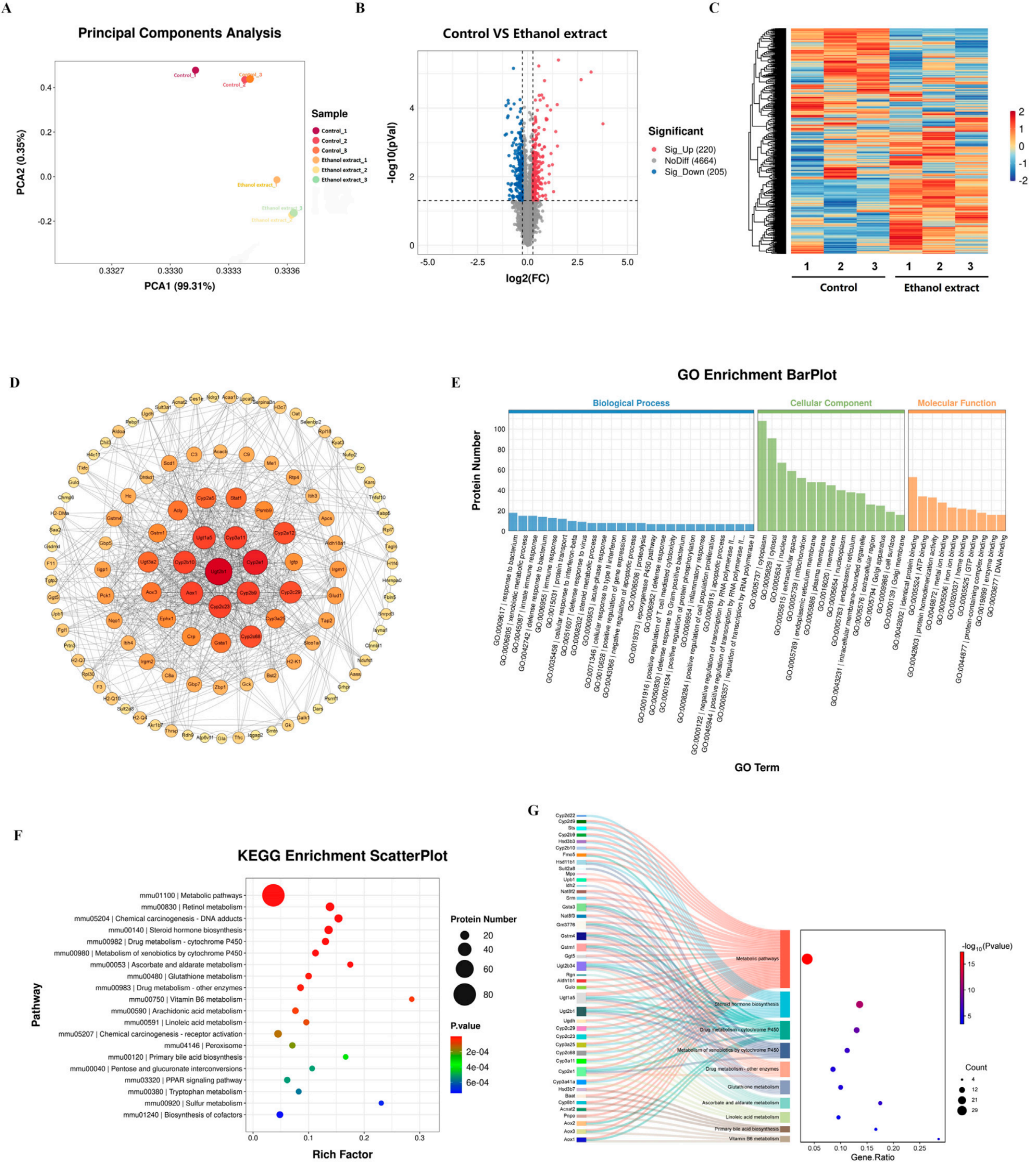
Proteomics analyses of liver tissue from mice treated with phosphate buffer saline or *Emilia sonchifolia* ethanol extract. **(A)** Principal components analysis to identify clusters of hepatic proteomes from mice treated with phosphate buffer saline or *Emilia sonchifolia* ethanol extract (n = 3). **(B)** Volcano plot of differentially expressed protein in the hepatic proteomics, in which the Bonferroni-adjusted -log_10_ (probability) is plotted against log_2_ (fold change). Significantly upregulated proteins are shown as red dots; significantly downregulated proteins, as blue dots. **(C)** Hierarchical cluster analysis of differentially expressed proteins between the liver tissues from control mice and mice treated with *Emilia sonchifolia* ethanol extract. **(D)** The protein-protein interaction (PPI) network was constructed using STRING (http://www.string-db.org) and Cytoscape software. Nodes represent proteins, while edges indicate interactions. The size and color of each node indicate the degree of connectivity, with larger and darker nodes representing higher degrees of interaction. **(E)** The GO enrichment Barplot for differentially expressed proteins in hepatic proteomics. The x-axis represents GO terms, while the y-axis indicates the number of proteins. Blue bars correspond to biological processes, green bars to cellular components, and orange bars to molecular functions. **(F)** The KEGG enrichment analysis of differentially expressed proteins in liver tissues from control mice and mice treated with *Emilia sonchifolia* ethanol extract. **(G)** The Sankey diagram illustrates the association between differentially expressed proteins (left) and KEGG pathways (right) identified in the hepatic proteomics analysis.

We first conducted proteomic sequencing and analysis of the livers from two groups of mice. The results showed significant differences in principal component analysis distances between control mice and the mice treated with a *E. sonchifolia* ethanol extract (Figure 3A). The results showed that the *E. sonchifolia* ethanol extract significantly altered the hepatic protein profile in mice. A total of 425 differentially expressed proteins (220 upregulated and 205 downregulated) with significantly distinct expression patterns before and after ethanol extract treatment were identified (Figures 3B,C). Among them, E9QAA8, G3X8P9 and Q9CWL8 were key differentially expressed proteins with the greatest fold changes, all of which displayed highly significant differences (Figure 3B; Table 2). The differentially expressed proteins were imported into the STRING database to construct a protein-protein interaction network. The corresponding TSV file was then exported and analyzed using Cytoscape software. Core genes were identified based on degree centrality, as shown in Figure 3D, with Ugt2b1 emerging as the most central gene. GO enrichment analysis revealed that the biological processes primarily involved response to bacterium, xenobiotic metabolic process, innate immune response, defense response to bacterium, and steroid metabolic process. The cellular components were mainly enriched in the cytoplasm and cytosol, while molecular functions were associated with identical protein binding, ATP binding, and protein homodimerization activity (Figure 3E). KEGG pathway analysis revealed significant changes in metabolic pathways, steroid hormone biosynthesis, drug metabolism-cytochrome P450, metabolism of xenobiotics by cytochrome P450, drug metabolism-other enzymes, and primary bile acid biosynthesis in the liver tissue of mice treated with the *E. sonchifolia* ethanol extract (Figure 3F). Changes in metabolic pathways are associated with the proteins Aox1, Cyp2c29, Cyp3a41a, Cyp2b9, Cyp2e1, Ugt2b1, Ugt1a5, Cyp2c23, Cyp2c68, Hsd3b7, Cyp2b10, Hsd3b3, Cyp3a11, Ugt2b34, Hsd11b1, Cyp3a25, etc. Changes in steroid hormone biosynthesis are linked to the proteins Cyp3a41a, Cyp2b10, Cyp2e1, Hsd3b3, Ugt2b1, Cyp3a11, Ugt1a5, Cyp2b9, Cyp2c68, Cyp3a25, Cyp2c23, Cyp2c29, Sts, Cyp2d9, Ugt2b3, Hsd11b1, and Cyp2d22. Alterations in drug metabolism through cytochrome P450, metabolism of xenobiotics by cytochrome P450, and drug metabolism by other enzymes involve the proteins Cyp2e1, Gstm1, Ugt2b1, Ugt1a5, Gstm4, Gm3776, and Ugt2b34. Additionally, changes in primary bile acid biosynthesis involve the proteins Acnat2, Cyp8b1, Baat, and Hsd3b7 (Figure 3G).

**TABLE 2:** The top 3 differential proteins.

Protein_name	Gene_name	FC	P_value	Q_value
E9QAA8	Gm4841	13.5889	0.000004	0.01145
G3X8P9	Aox1	8.9977	0.000006	0.01145
Q9CWL8	Ctnnbl1	6.3707	0.000007	0.01145

**FIGURE 4:**
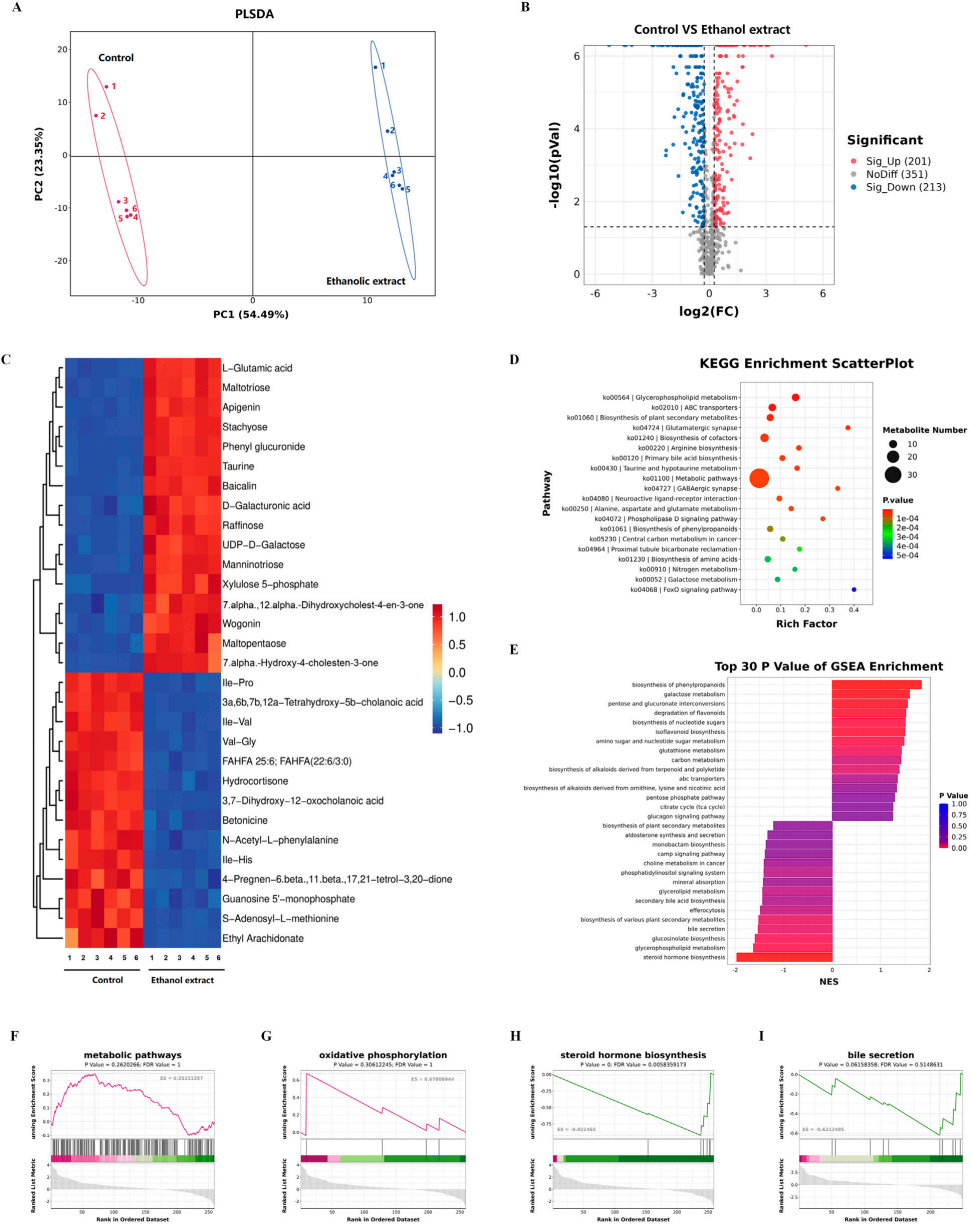
Metabolomics analyses of liver tissue from mice treated with phosphate buffer saline or *Emilia sonchifolia* ethanol extract. **(A)** Partial least squares discriminant analysis to identify clusters of hepatic metabolomes from mice treated with phosphate buffer saline or *Emilia sonchifolia* ethanol extract (n = 3). **(B)** Volcano plot of differentially expressed metabolites in the hepatic proteomics, in which the Bonferroni-adjusted -log_10_ (probability) is plotted against log_2_ (fold change). Significantly upregulated metabolites are shown as red dots; significantly downregulated metabolites, as blue dots. **(C)** Hierarchical cluster analysis of the top 30 differentially expressed metabolites between the liver tissues from control mice and mice treated with *Emilia sonchifolia* ethanol extract. **(D)** The KEGG enrichment analysis of differentially expressed metabolites in liver tissues from control mice and mice treated with *Emilia sonchifolia* ethanol extract. **(E)** Gene set enrichment analysis of differential metabolites between the liver tissues from control mice and treated with *Emilia sonchifolia* ethanol extract. **(F–I)** Gene set enrichment analysis, indicating enrichment of processes related to response to metabolic pathways **(F)**, oxidative phosphorylation **(G)**, steroid hormone biosynthesis **(H)** and bile secretion **(I)** in the metabolomes of liver tissues from mice treated with *Emilia sonchifolia* ethanol extract compared to the control mice.

Then, we conducted metabolomic sequencing and analysis of the livers from two groups of mice. The results showed significant differences in partial least squares discriminant analysis distances between control mice and the mice treated with a *Emilia sonchifolia* ethanol extract (Figure 4A). This result indicates that the *E. sonchifolia* ethanol extract significantly alters the hepatic metabolic profile in mice. A total of 414 differentially expressed proteins (201 upregulated and 213 downregulated) with significantly distinct expression patterns before and after ethanol extract treatment were identified (Figure 4B). The expression levels of differential metabolites, including taurine, 7α,12α-dihydroxycholest-4-en-3-one, and 7α-hydroxy-4-cholesten-3-one were significantly upregulated (Figure 4C). KEGG pathway analysis revealed significant changes in metabolic pathways, primary bile acid biosynthesis, and Taurine and hypotaurine metabolism in the liver tissue of mice treated with the *E. sonchifolia* ethanol extract (Figure 4D). Further gene set enrichment analysis enrichment analysis revealed significant activation of metabolic pathways and oxidative phosphorylation, while steroid hormone biosynthesis and bile secretion pathways were significantly suppressed (Figures 4E–I). In phase I drug metabolism, the toxic pyrrolizidine alkaloids (PAs) from *E. sonchifolia* can be oxidised by CYP450 enzymes and eventually form adducts, which can cause hepatotoxicity (Almazroo et al., 2017). And the blockage of glucuronidation process in phase II drug metabolism can lead to disorders of bile acid metabolism, which in turn causes liver injury (Hu et al., 2014; King et al., 2000). Steroid hormones can alleviate cholestasis by inhibiting inflammatory responses (Payne and Freishtat, 2012), and when steroid hormone biosynthesis is impaired, cholestasis and subsequent hepatic damage ensue. Enhanced primary bile acid biosynthesis further increases bile acid production, thereby exacerbating liver injury (Cai and Boyer, 2021). During this process, metabolites such as 7α-hydroxy-4-cholesten-3-one, 7α,12α-dihydroxycholest-4-en-3-one, and taurine were found to be upregulated. Thus, it can be concluded that under the co-regulation of phase I drug metabolism as a major pathway, phase II drug metabolism, steroid hormone biosynthesis and primary bile acid biosynthesis as a minor pathway, cholestasis is formed. This condition further induces oxidative stress, ultimately leading to the development of hepatotoxicity.

**FIGURE 5:**
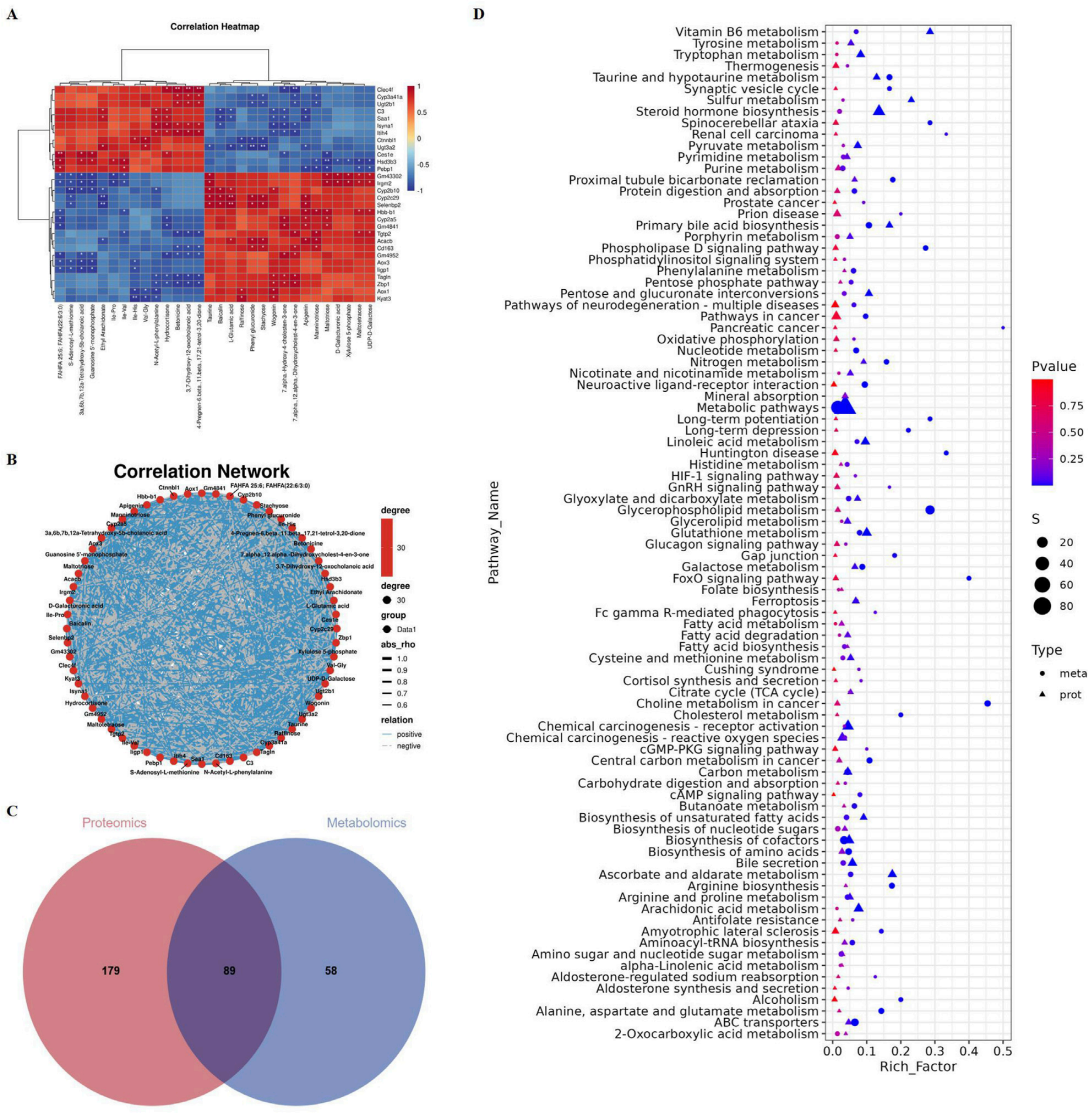
Proteomics and metabolomics integrated analysis of liver tissue from mice treated with phosphate buffer saline or *Emilia sonchifolia* ethanol extract. **(A)** Correlation clustering heat map of proteomics and metabolomics. **(B)** Correlation network map of proteomics and metabolomics. **(C)** Venn diagram of significantly enriched KEGG pathways in proteome and metabolome. **(D)** Bubble plot showed the 89 KEGG pathways that were co-enriched in proteome and metabolome.

Next, we conducted an integrated analysis of proteomics and metabolomics. There is a significant correlation among these proteins and metabolites (Figures 5A,B). Correlation clustering heat map and network map revealed that the expression of the protein Cyp2c29 was upregulated, while Ugt2b1, Cyp3a41a, and Hsd3b3 were downregulated. These proteins are closely associated with pathways such as drug metabolism and steroid hormone biosynthesis (Sunoqrot et al., 2024). Additionally, the metabolites taurine, 7α,12α-dihydroxycholest-4-en-3-one, and 7α-hydroxy-4-cholesten-3-one were significantly upregulated, which are closely linked to primary bile acid biosynthesis (Luo et al., 2024; Yin et al., 2024). The Venn diagram indicated that 89 pathways were commonly enriched in both the proteomics and metabolomics analyses (Figure 5C). The significantly enriched pathways included metabolic pathways, steroid hormone biosynthesis, and primary bile acid biosynthesis (Figure 5D).

**FIGURE 6:**
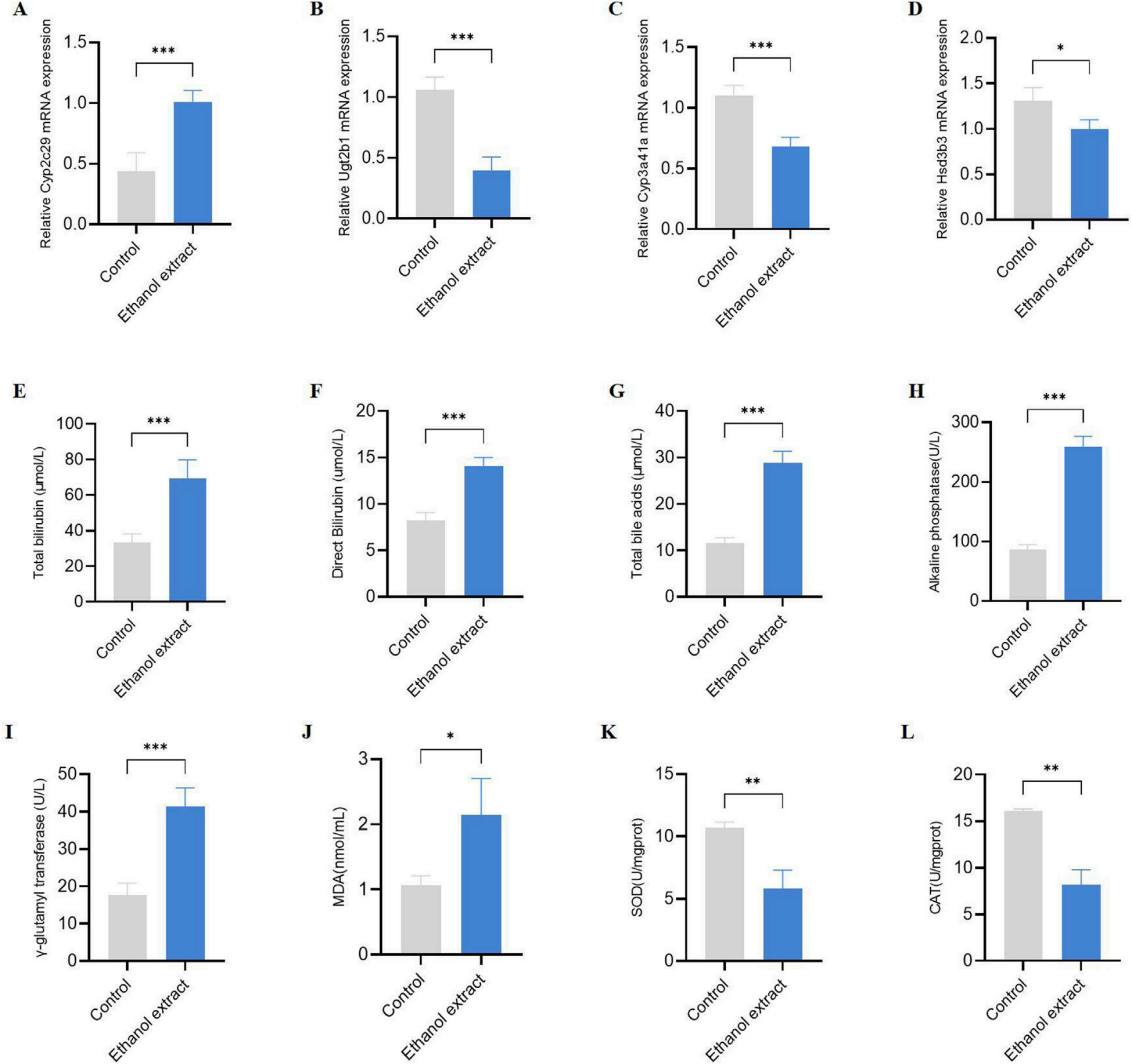
Validation of the hepatotoxicity mechanism of *Emilia sonchifolia* ethanol extract. **(A-D)** The relative mRNA expression of key differentially expressed genes (*Cyp2c29*, *Cyp3a41a, Ugt2b1*, and *Hsd3b3*) in liver tissue from mice treated with phosphate buffer saline or *Emilia sonchifolia* ethanol extract. **(E-I)** Quantification of total bilirubin (E), direct bilirubin (F), total bile acids (G), alkaline phosphatase (H), and γ-glutamyl transferase (I) in serum from the mice treated with phosphate buffer saline or *Emilia sonchifolia* ethanol extract. **(J-L)** Quantification of malondialdehyde (J), superoxide dismutase (K) and catalase (L) in liver tissue from mice treated with phosphate buffer saline or *Emilia sonchifolia* ethanol extract. Data are presented as mean ± SEM (n = 4). *p < 0.05, ***p < 0.001 by unpaired t-test.

Taken together, we hypothesized that the hepatotoxicity induced by *E. sonchifolia* ethanol extract is associated with cholestasis and oxidative stress mediated by *Cyp2c29*, *Cyp3a41a*, *Ugt2b1* and *Hsd3b3*. We have validated the above hypothesis, and the results show that in mice treated with *E. sonchifolia* ethanol extract, the gene expression level of *Cyp2c29* was upregulated, while the expression levels of *Ugt2b1*, *Cyp3a41a* and *Hsd3b3* were downregulated (Figures 6A–D). Meanwhile, compared to the control group, mice treated with *E. sonchifolia* ethanol extract exhibited significantly elevated serum levels of total bilirubin, direct bilirubin, total bile acids, alkaline phosphatase, and γ-glutamyl transpeptidase (Figures 6E–I). Furthermore, in the livers of mice treated with *E. sonchifolia* ethanol extract, the malondialdehyde level was increased, whereas the levels of superoxide dismutase and catalase were significantly decreased (Figures 6J–L).

## 4 Discussion

*Emilia sonchifolia* is a plant that possesses both medicinal and dietary applications (George and Kuttan, 2019; Jeeno et al., 2023). The PAs of *E. sonchifolia* are natural toxins, which can cause significant hepatomegalia, live venoocclusive disease, hepato-carcinogenicity, neurotoxicity, mutagenicity and embryotoxicity (Luo et al., 2019). *Emilia sonchifolia* is typically prepared using either water or ethanol extraction (An et al., 2025; Liu et al., 2025). Although our previous study has characterized the hepatotoxicity of the water extract of *E. sonchifolia*, the pathological mechanisms of its ethanol extract remain to be elucidated. In this study, we evaluated *E. sonchifolia* ethanol extract’s hepatotoxicity and investigated its mechanism by proteomics and metabolomics analysis. The results revealed that the hepatotoxicity induced by *E. sonchifolia* ethanol extract is associated with cholestasis and oxidative stress mediated under the co-regulation of phase I drug metabolism as a major pathway, phase Ⅱ drug metabolism, steroid hormone biosynthesis and primary bile acid biosynthesis as a minor pathway, which had some similarities with the mechanism of action of water extract. To the best of our knowledge, this study represents the first systematic investigation into the mechanism of hepatotoxicity induced by *E. sonchifolia* ethanol extract.

Currently, the evaluation of *E. sonchifolia* dosage remains inadequate. In this study, an acute toxicity preliminary screening study was performed by orally administering *E. sonchifolia* ethanol extract to mice at doses ranging from 0 to 33.6 g/kg. The results revealed that a dose of 12.0 g/kg induced acute liver failure and mortality in mice. At doses equal to or exceeding 24.0 g/kg, the survival rate was 0%. In contrast, doses of 8.6 g/kg or lower resulted in a 100% survival rate, demonstrating dose-dependent acute hepatotoxicity. Notably, doses below 8.6 g/kg did not result in acute liver failure.

We further investigated the delayed hepatotoxicity of *E. sonchifolia* ethanol extract at a dose of 8.6 g/kg. The results demonstrated that administering 8.6 g/kg of *E. sonchifolia* ethanol extract to mice for 14 days led to a significant increase in hepatocyte vacuolation and nuclear pyknosis. Additionally, the serum levels of alanine aminotransferase and aspartate aminotransferase were significantly elevated. The cytoplasm of hepatocytes contains various biochemical enzymes, and the levels of serum liver biomarker enzymes, such as alanine aminotransferase and aspartate aminotransferase, are essential parameters for the biochemical assessment of potential hepatotoxic effects (Tamber et al., 2023). When the liver is damaged, these enzymes are released from hepatocytes into the bloodstream, resulting in elevated serum levels (Ceriotti et al., 2010; Robles-Diaz et al., 2015). These findings indicate that *E. sonchifolia* ethanol extract exhibits delayed hepatotoxicity in mice.

Furthermore, we further investigated the mechanisms underlying the hepatotoxicity induced by *E. sonchifolia* ethanol extract using integrated proteomics and metabolomics analysis. The results demonstrated that the ethanol extract significantly altered the proteomic and metabolic profiles in the livers of treated mice compared to controls. Analysis of the proteomics and metabolomics data revealed that the hepatotoxic effects of *E. sonchifolia* ethanol extract are closely associated with disruptions in drug metabolism, steroid hormone biosynthesis, and primary bile acid biosynthesis. Moreover, the integrated analysis further indicated that the hepatotoxic mechanism involves cholestasis and oxidative stress mediated by *Cyp2c29*, *Ugt2b1*, *Cyp3a41a*, and *Hsd3b3*.

The term “drug metabolism” refers to the enzymatic transformation of chemicals from one chemical moiety to another, involving two types of reactions: Phase I and Phase II (King et al., 2000). The most common Phase I drug-metabolizing enzymes belong to the CYP450 superfamily (Almazroo et al., 2017; Zhao et al., 2021). In humans, CYP450 enzymes are distributed across various tissues and organs, including peripheral blood cells, platelets, the aorta, adrenal glands, adipose tissue, nasal and vaginal tissues, seminal vesicles, brain, lungs, kidneys, gut, and liver. Among these, the liver and small intestine play the most significant roles in the overall metabolism and elimination of drugs (Louisse et al., 2022). Among the CYP450 enzymes, CYP1, CYP2, and CYP3 are the most abundant (Almazroo et al., 2017). In our study, the *Cyp2C29* was upregulated, which indicated that *E. sonchifolia* extract metabolism were enhanced, leading to increased metabolites. The metabolites can be either pharmacologically active or inactive. PAs in *E. sonchifolia* are oxidized by cytochrome P450 enzymes, mainly in the liver, to 6,7-dihydro-7-hydroxy-1-(hydroxymethyl)-5H-pyrrolizine - DHP (pyrrolicester), a strong electrophile that can form adducts with biological macromolecules, such as protein and DNA. This bioactive metabolite is considered the main reason for PAs toxicity. Thus, we deduced that the hepatotoxicity of *E. sonchifolia* extracts was related to increase of *Cyp2C29* expression. During phase II drug metabolism, the drugs or metabolites from phase I reactions are enzymatically conjugated with hydrophilic endogenous compounds by transferase enzymes. The most common phase II drug-metabolizing enzymes include UDP-glucuronosyltransferases, sulfotransferases, N-acetyltransferases, glutathione S-transferases, thiopurine S-methyltransferases, and catechol O-methyltransferases. In humans, three subfamilies of UDP-glucuronosyltransferases (UGT), namely, UGT1A, UGT2A, and UGT2B, are primarily responsible for glucuronidation (Hu et al., 2014). Glucuronidation is a key metabolic pathway for many small endogenous and exogenous lipophilic compounds, including bilirubin, steroid hormones, bile acids, carcinogens, and therapeutic drugs (King et al., 2000). In our study, *Ugt2b1* was downregulated, which demonstrated that glucuronidation was disturbed, resulting in metabolic disorders of bile acids. It led to cholestasis, which can cause liver damage.

Previous studies have shown that steroid hormones can inhibit inflammation via Mek1 and Erk1, which are members of the MAPK pathway (Payne and Freishtat, 2012). Moreover, the results from Ibone Labiano et al.'s study demonstrate that TREM-2 plays a protective role in cholestasis by acting as a negative regulator of inflammation (Labiano et al., 2022). In other words, steroid hormones can suppress cholestasis by inhibiting inflammation. The synthesis of steroid hormones involves several enzymes, including cytochrome P450 enzymes. *Cyp3a41a* is a cytochrome P450 enzyme in mice that is homologous to human CYP3A4 and CYP3A5. These enzymes play a crucial role in the biosynthesis of steroid hormones, primarily responsible for catalyzing the multi-step oxidative reactions that convert cholesterol into various steroid hormones (Hanuko and glu, 1992). Hsd3b3 is an enzyme with both 3-beta-hydroxysteroid-delta5-dehydrogenase activity and steroid delta-isomerase activity. It is involved in several processes, including hippocampal development, response to corticosterone, and steroid hormone biosynthesis. Hsd3b3 is located in the inner mitochondrial membrane and is expressed in the adrenal glands, liver, and medullary region of the testes. Additionally, its human homologs, HSD3B1 and HSD3B2, are associated with hypertension and hypospadias, further highlighting the importance of *Hsd3b3* and its human counterparts in steroid hormone biosynthesis. Furthermore, hydroxysteroid dehydrogenases (HSDs) catalyse the oxidation/reduction of hydroxy (-OH)/oxo groups of steroids. This reaction type contributes fundamental steps in the biosynthesis of vertebrate steroid hormones (Shafqat et al., 2003). *Hsd3b3* plays a central role in steroid hormone biosynthesis, which shares cholesterol as a common precursor with bile acid synthesis (Cai et al., 2022; Chiang, 2004). Downregulation of *Hsd3b3* may decrease the flux of cholesterol toward steroid hormone formation and concomitantly enhance its conversion into bile acids, thereby promoting bile acid accumulation and cholestasis (Chiang and Ferrell, 2020; Song et al., 2025). Steroid hormones, particularly glucocorticoids, have been reported to alleviate cholestasis by suppressing inflammatory responses and regulating bile acid transporters (Halilbasic et al., 2013; Trauner et al., 2017). Hence, impaired steroidogenesis resulting from *Hsd3b3* inhibition may further exacerbate bile acid–induced liver injury (Song et al., 2025; Zeng et al., 2023). Taken together, *Hsd3b3* plays a critical role in steroid hormone synthesis, particularly in catalyzing key steps in the steroidogenesis pathway (Vagnerová et al., 2024). In our study, the expression of *Cyp3a41a* and *Hsd3b3* was downregulated. Therefore, we speculate that *E. sonchifolia* ethanol extract inhibits steroid hormone biosynthesis, thereby suppressing bile secretion and ultimately leading to cholestasis.

The accumulation of bile acids leads to cholestatic liver diseases, which serve as markers for liver injury metabolites (Zeng et al., 2023). 7α-Hydroxy-4-cholesten-3-one is a key intermediate in the cholesterol-to-primary bile acid synthesis pathway (Synthesis of Cholesterol from 7α-Hydroxy-4-cholesten-3-one in the Intestine) and plays a role in bile acid synthesis (Gälman et al., 2003). 7α,12α-Dihydroxycholest-4-en-3-one is another crucial intermediate in the bile acid biosynthesis pathway and serves as an important biomarker for assessing the levels of hepatic bile acid synthesis (Ogawa et al., 2013). Taurine is an essential component in bile acid metabolism. After primary bile acids are synthesized in the liver, they conjugate with taurine or glycine to form bile acid conjugates. This conjugation is crucial for the solubility and excretion of bile acids (Miyazaki et al., 2023). Physiologically, taurine conjugation increases bile acid solubility, lowers membranolytic toxicity, and promotes biliary excretion - mechanisms that protect hepatocytes (Duszka, 2022; Hofmann and Hagey, 2008). Following hepatotoxicity induced by *E. sonchifolia* ethanol extract, taurine levels were significantly elevated. Given that taurine conjugation enhances bile acid solubility and facilitates excretion, this increase in taurine likely reflects a compensatory response of the organism aiming to promote bile acid detoxification and protect against bile acid–induced liver injury. However, under conditions of excessive bile acid synthesis or impaired canalicular secretion, the observed elevation in free taurine may represent an adaptive attempt to improve conjugation and excretion that is insufficient to offset the accumulating load (Pablo et al., 2017). Although taurine elevation appears to be a compensatory response aiming to enhance bile acid conjugation and detoxification, the persistent accumulation of bile acids despite this adaptation suggests that such compensation is insufficient to prevent cholestasis. In our study, the *E. sonchifolia* ethanol extract upregulated metabolites such as 7α-Hydroxy-4-cholesten-3-one, 7α,12α-Dihydroxycholest-4-en-3-one, and Taurine, leading to an increase in primary bile acids and consequently resulting in cholestasis.

Taken together, phase II drug metabolism enzymes, steroid hormones, and primary bile acids are all associated with cholestasis. Total bilirubin, direct bilirubin, total bile acids, alkaline phosphatase, and γ-glutamyl transferase are important biomarkers for verifying cholestasis (Ozgen et al., 2022; Tang et al., 2024). We validated these markers, and the results were consistent with our hypothesis. Oxidative stress is a key factor that accompanies liver injury in cholestasis (Sha et al., 2021; Yuan et al., 2018). Meanwhile, oxidative stress can promote lipid peroxidation, leading to an increase in malondialdehyde (a lipid peroxidation byproduct) levels and a decrease in superoxide dismutase and catalase (antioxidant enzymes) (Del et al., 2005; Frijhoff et al., 2015; Wang et al., 2024). Similar results were observed in our study, where *E. sonchifolia* ethanol extract increased malondialdehyde levels and decreased superoxide dismutase and catalase activity.

In summary, it can be concluded that the ethanol extract of *E. sonchifolia* can upregulate the expression of *Cyp2C29* in phase I drug metabolism, thereby leading to an increase in toxic products and inducing hepatotoxicity, which may represent the major pathway responsible for the early hepatotoxicity caused by *E. sonchifolia*. Secondly, the ethanol extract of *E. sonchifolia* can downregulate the expression of *Ugt2b1* in phase II drug metabolism, resulting in bile acid metabolic disorders. At the same time, the extract acts on steroid hormone biosynthesis, downregulating the expression of *Cyp3a41a* and *Hsd3b3*, which suppresses bile excretion and causes cholestasis. In addition, the extract affects the primary bile acid biosynthesis pathway, leading to increased expression of metabolites such as 7α-Hydroxy-4-cholesten-3-one, 7α,12α-Dihydroxycholest-4-en-3-one, and Taurine, which in turn promotes bile acid production. Therefore, phase II drug metabolism, steroid hormone biosynthesis, and primary bile acid biosynthesis serve as secondary pathways through which the ethanol extract of *E. sonchifolia* collectively contributes to cholestasis, while cholestasis further induces oxidative stress, ultimately leading to hepatotoxicity.

In conclusion, the hepatotoxicity mechanisms of *E. sonchifolia* ethanol extract involve two key aspects: (1) the extract increases the production of toxic metabolites in the liver by enhancing phase I drug-metabolizing enzymes, particularly CYP450, and (2) the extract induces oxidative stress in the liver by promoting cholestasis. This study suggests two potential strategies for addressing liver damage caused by *E. sonchifolia* ethanol extract. First, drugs that decrease the activity of phase I drug-metabolizing enzymes, particularly CYP450, could help reduce the production of toxic metabolites. Second, future research could focus on identifying drugs that regulate bile metabolism by enhancing phase II drug metabolism enzymes, inhibiting steroid hormone biosynthesis, and modulating primary bile acid synthesis to reduce or eliminate the hepatotoxic effects. This study compensates for the limitation of investigating only the hepatotoxicity mechanism of the water extract of *E. sonchifolia*, and provides a more comprehensive theoretical basis for the development, utilization, and safety evaluation of *E. sonchifolia*.

## Data Availability

The data presented in the study are deposited in the Metabolights repository (accession number MTBLS13237) and in the iProX database (accession number PXD070070).

## Abbreviations

ELISA, Enzyme-Linked Immunosorbent Assay; GO, Gene Ontology; KEGG, Kyoto Encyclopedia of Genes and Genomes; qRT-PCR, Quantitative Reverse Transcription Polymerase Chain Reaction.
